# Myeloid-Derived Suppressor Cells in Bladder Cancer: An Emerging Target

**DOI:** 10.3390/cells13211779

**Published:** 2024-10-27

**Authors:** Clément Klein, Samy Mebroukine, Mathilde Madéry, Alexandra Moisand, Thomas Boyer, Nicolas Larmonier, Grégoire Robert, Charlotte Domblides

**Affiliations:** 1 CNRS UMR 5164, ImmunoConcEpT, Biological and Medical Sciences Department, University of Bordeaux, 146 rue Léo Saignat, 33000 Bordeaux, France; clement.klein@chu-bordeaux.fr (C.K.); samy.mebroukine@u-bordeaux.fr (S.M.); mathilde.madery@u-bordeaux.fr (M.M.); alexandra.moisand@u-bordeaux.fr (A.M.); thomas.boyer@u-bordeaux.fr (T.B.); nicolas.larmonier@u-bordeaux.fr (N.L.); gregoire.robert@chu-bordeaux.fr (G.R.); 2 Department of Urology, University Hospital of Bordeaux, 33000 Bordeaux, France; 3 Department of Biological and Medical Sciences, University of Bordeaux, 33000 Bordeaux, France; 4 Department of Medical Oncology, University Hospital of Bordeaux, 33000 Bordeaux, France

**Keywords:** myeloid-derived suppressor cells, bladder cancer, tumor microenvironment, immune inhibition, biomarker, immunotherapy

## Abstract

Bladder cancer remains a prevalent and challenging malignancy. Myeloid-derived suppressor cells (MDSCs) have emerged as key contributors to the immunosuppressive tumor microenvironment, facilitating tumor progression, immune evasion, and resistance to therapies. This review explores the role of MDSC in bladder cancer, highlighting their involvement in immune regulation; tumor progression; and resistance to therapies such as bacillus Calmette–Guérin (BCG) therapy, chemotherapy, and immune checkpoint inhibitors (ICIs). We also discuss their potential as biomarkers and therapeutic targets, with current evidence suggesting that targeting MDSCs, either alone or in combination with existing treatments such as BCG and ICIs, may enhance anti-tumor immunity and improve clinical outcomes. However,, challenges remain, particularly regarding the identification and therapeutic modulation of MDSC subpopulations. Further research is warranted to fully elucidate their role in bladder cancer and to optimize MDSC-targeted therapies.

## 1. Introduction

### Epidemiology and Prognosis

Bladder cancer (BC) is the tenth most commonly diagnosed cancer worldwide, with an age-standardized mortality rate of 3.3 per 100,000 men per year versus 0.86 for women. The incidence and mortality of BC have decreased in some regions, likely due to the reduction of causative factors such as smoking, attributed to prevention measures promoted by the World Health Organization (WHO) [1].

At the time of diagnosis, approximately 75% of patients present with disease limited to the mucosa (stage Ta, CIS) or submucosa (stage T1), typically classified as non-muscle-invasive bladder cancer (NMIBC). These patients have high rates of recurrence due to long-term survival in many cases and a lower risk of cancer-specific mortality compared to patients with muscle-invasive bladder cancer (MIBC), which is characterized by tumor cell invasion into the muscular layer of the urothelium.

The oncological prognosis of NMIBC is primarily associated with the risk of progression to MIBC (10–30% of the cases) [2,3]. Several prognostic models specific to patient populations have been developed to predict this risk [1]. In the case of MIBC, the prognosis worsens with advanced stage and remains poor despite treatment advances, with a 5-year relative survival rate of 50–70% for localized disease and only 5–15% for metastatic disease [4]. Thus, limiting the risk of NMIBC progression and relapse remains a critical aim to improve patient prognosis. Several treatments have been developed with mitigated success, and there is a crucial need to develop innovative therapeutic strategies.

## 2. Current Therapeutic Management

### 2.1. Non-Muscle-Invasive Bladder Cancer (NMIBC)

According to international guidelines, the standard treatment for NMIBC involves transurethral resection of the bladder (TURB) followed by intravesical instillations of bacillus Calmette–Guérin (BCG) or chemotherapy depending on the prognostic group [1]. BCG has been used for over 30 years to treat NMIBC, reducing the risk of recurrence (32%) and progression (27%) compared to observation or intravesical chemotherapy [5,6]. Despite extensive clinical experience with BCG, the mechanisms of its therapeutic effect remain under investigation [7]. Besides its direct cytotoxic effect against cancer cells, BCG modulates the immune system by interacting with pattern recognition receptors (PRRs) on antigen-presenting cells (APCs), inducing their activation and the presentation of tumor antigens to CD4^+^ and CD8^+^ T cells, leading to an antitumor immune response. However, many patients do not respond to this treatment, relapsing shortly afterwards, or they cannot tolerate the standard dose. In addition, the supply of the BCG Connaught strain remains uncertain.

### 2.2. Muscle-Invasive Bladder Cancer (MIBC)

The standard treatment for localized MIBC consists of cisplatin-based neoadjuvant chemotherapy (NAC) followed by radical cystectomy with locoregional lymph node removal. Radical cystectomy alone allows for a 5-year survival of approximately 50% of patients [4]. Since the early 1980s, neoadjuvant cisplatin-based chemotherapy has been used to increase the pathological complete response (pCR) rate after cystectomy, improving overall survival by 5% to 8% compared to patients treated with cystectomy alone [8].

### 2.3. Locally Advanced and Metastatic Disease

For locally advanced disease with regional lymph node involvement confirmed by imaging (cN+), without distant metastases (M0), the standard of care remains cisplatin-based chemotherapy induction, potentially followed by cystectomy based on the response [4]. In metastatic disease, platinum-based chemotherapy, primarily cisplatin, remains the first-line treatment, although the clinical efficacy of immune checkpoint inhibitors (ICIs) offers promising new therapeutic perspectives.

## 3. Contribution of Immunotherapy in Bladder Cancer

### 3.1. Non-Muscle-Invasive Bladder Cancer (NMIBC)

As mentioned, many patients do not respond to BCG therapy, relapse shortly after treatment, or cannot tolerate the standard dose. For these patients, radical cystectomy together with pelvic lymph node dissection is recommended. However, radical cystectomy is the most morbid surgical procedure in urology, with perioperative complication rates reaching more than 60% of patients. Furthermore, perioperative mortality is also concerning, with estimated rates between 1% and 3%. The 90-day perioperative mortality rate increases to 5%, 7%, and 11% in patients aged 65, 75, and 80 or older, respectively [9].

Conservative treatments with intravesical instillations or intravesical thermo-chemotherapy are considered possible alternatives in cases of refusal or ineligibility for radical surgery, but with worsened oncological outcomes [1]. Thus, for this indication of high-risk NMIBC, numerous treatments are being tested (immunotherapy, gene therapy, intravesical chemotherapy), either in combination with or as an alternative to BCG in BCG-naive patients, and also in cases of early recurrence after BCG therapy (BCG refractory).

Among patients with early recurrence after BCG therapy, the results of KEYNOTE-057 (NCT02625961) and SWOG S1605 (NCT02844816) studies demonstrated limited efficacy of Pembrolizumab and Atezolizumab with event-free survivals of 43% and 30% at 6 months, respectively [10,11]. These immunomodulatory antibodies target the PD-1/PD-L1 axis, which is a key inhibitory immune checkpoint. By blocking the interaction between PD-1 on T cells and PD-L1 on tumor cells, they release the brakes on the immune system, allowing T cells to remain active. This results in enhanced T-cell-mediated tumor cell killing and an improved anti-tumor immune response.

Among BCG-naïve patients, several clinical trials are ongoing to test combinations of BCG with PD-L1 or PD-1 antibodies such as Atezolizumab (ALBAN, NCT03799835), Durvalumab (POTOMAC, NCT03528694), Sasanlimab (CREST, NCT04165317), and Pembrolizumab (KEYNOTE-676, NCT03711032), with pending results.

### 3.2. Muscle-Invasive Bladder Cancer (MIBC)

For MIBC, ICIs have been evaluated in both neoadjuvant and adjuvant settings. In the neoadjuvant setting, PD-1/PD-L1 inhibitors have been tested as monotherapy or in combination with CTLA-4 inhibitors or chemotherapy. The PURE-01 and ABACUS trials reported pCR rates of 42% and 31%, respectively, for Pembrolizumab and Atezolizumab monotherapy [12,13]. Combinations with chemotherapy also showed promising results, with pCR rates up to 41%. Additionally, the NIAGARA study recently reported a pCR rate of 37.3% with Durvalumab in combination with chemotherapy (gemcitabine–cisplatin) and 27.5% in the control group, highlighting the potential efficacy of this approach [14].

In the adjuvant setting, phase III trials CheckMate 274 and IMvigor010 evaluated Nivolumab and Atezolizumab, respectively, showing significant improvements in disease-free survival (DFS) with Nivolumab for patients at high risk of recurrence after surgery [15,16].

### 3.3. Metastatic Disease

For metastatic disease, ICIs have demonstrated similar efficacy and safety in patients with disease progression after platinum-based chemotherapy. The JAVELIN Bladder 100 study showed improved overall survival with Avelumab maintenance after initial platinum–gemcitabine chemotherapy, compared to best supportive care (OS = 21.4 vs. 14.3 months, respectively; HR, 0.69; 95% [CI], 0.56–0.86; *p* = 0.001) [17]. Pembrolizumab also showed significant overall survival improvement as a second-line treatment compared to chemotherapy (median 10.3 vs. 7.4 months, respectively; HR, 0.73; 95% CI, 0.59 to 0.91; *p* = 0.002) [18] and is a new option as a first-line treatment in association with Enfortumab–Vedotin [4,19].

## 4. Low Response Rates to Immunotherapy: How to Improve Patients’ Outcomes?

### 4.1. Lack of Biomarkers

Even though immunotherapy shows interesting responses, some patients do not respond, and many relapse. PD-L1 status has been developed as a predictive biomarker for immunotherapy but lacks accuracy, and immunotherapy response remains hard to predict at any treatment stage. Several studies have therefore attempted to identify biomarkers to predict response to immunotherapy, but none have shown objective value for clinical use [20,21].

### 4.2. Targeting Other Components Within the Tumor Microenvironment

While some patients do not respond to BCG or ICI therapies, it is becoming clear that ICI treatment is not a one-size-fits-all approach. Several studies suggest that its efficacy is highly dependent upon tumor characteristics and its associated immune microenvironment.

Intrinsic resistance to ICI can arise from the tumor itself, where genetic alterations or low mutational burden may limit the presentation of neoantigens, reducing the ability of immune cells to recognize and attack the tumor (immunoselection) [22]. On the other hand, resistance linked to the tumor microenvironment, which is induced by tumor cells (immunosubversion or tolerance), involves complex interactions with immune cells. As a result, lymphocyte exhaustion as well as the presence of immune inhibitory cells, characterized by the overexpression of immune checkpoint molecules like PD-1, are only partially captured by PD-L1 status, highlighting the limitations of using PD-L1 as a sole predictive marker. Moreover, various immunosuppressive cells, such as regulatory T cells (Tregs), tumor-associated macrophages (TAMs), and myeloid-derived suppressor cells (MDSCs), contribute to the induction of an immune-suppressive niche that hinders anti-tumor immunity [23]. Additionally, stromal cells, including cancer-associated fibroblasts, can form physical and biochemical barriers, preventing immune cell infiltration and sustaining a pro-tumorigenic environment. Addressing both intrinsic tumor mechanisms and microenvironmental factors is crucial for improving response rates to ICI.

## 5. Immunosuppressive Tumor Microenvironment and MDSCs: What Is Known?

Compelling evidence shows that multiple elements of the immune system critically contribute to cancer development and dissemination, exerting competing pro- and anti- tumor effects. The outcome of interactions between cancer and immune cells is dictated by a balance between inducing and maintaining potent effector immune responses and triggering numerous escape mechanisms by malignant cells. In this context, many immunoinhibitory networks have been described, leading to the establishment of a highly immunosuppressive tumor microenvironment. The expansion and promotion of suppressive immune cells, particularly myeloid-derived cells, significantly contribute to these immune escape mechanisms in many cancers.

MDSCs have been the focus of extensive research and have become a central element in oncology due to their critical role in the tumor-promoting landscape, their potential to serve as predictive biomarkers, and their therapeutic targetability. However, a common mistake in this field is considering these cells as a homogeneous subset.

Indeed, MDSCs are characterized as a morphologically, phenotypically, and functionally heterogeneous population of cells of myeloid origin, blocked in their differentiation at an immature stage [24]. Three main subpopulations of MDSCs have been identified in humans: polymorphonuclear MDSCs (*PMN-MDSCs*), resembling neutrophils; monocytic MDSCs (*M-MDSCs*), sharing features with monocytes; and early-stage MDSCs (*e-MDSCs*), which are at an even earlier stage of development (with emerging but not fully defined immunosuppressive capabilities). Furthermore, uncommitted MDSCs (UnC-MDSC) have also been described in the literature (Table 1).

Human MDSCs express CD33 and CD11b but lack maturation markers like HLA-DR, while in mice, the phenotypic characteristics of MDSCs differ slightly in terms of markers [25,26]. In addition, the specific markers for MDSCs are not clearly established, making their precise identification challenging. It is particularly difficult to distinguish MDSCs from polymorphonuclear neutrophils (PMNs) or M2 macrophages due to phenotypic similarities and functional overlaps [27].

**Table 1:** Phenotypic characteristics to identify cells as MDSC in mouse and human. PBMC, peripheral blood mononuclear cell. [Table adapted from [26]].

Type of MDSC	Mouse	Human (in PBMC Fraction)
Total (T)-MDSC	-	CD33^+^ CD11b^+^ HLA-DR^−/low^
PMN-MDSC	CD11b^+^ GR-1^+^	CD33^mid^ CD11b^+^ HLA-DR^−^ CD14^−^ CD15^+^ LOX-1^+^
M-MDSC	CD11b^+^ Ly6C^low^ Ly6G^+^	CD33^+^ CD11b^+^ HLA-DR^−/low^ CD14^+^ CD15^−^
UnC-MDSC	CD11b^+^ Gr-1^int^ Ly6G^low^ Ly6C^low^	CD33^+^ CD11b^+^ HLA-DR^−^ CD14^−^ CD15^+^
e-MDSC	CD11b^+^ Ly6C^hi^ Ly6G^−^	CD33^+^ CD11b^+^ HLA-DR^−/low^ Lin^−^

The mechanisms underlying the induction, expansion, and homing of MDSCs to the tumor site remain unclear. However, it is widely accepted that MDSCs primarily derive from hematopoietic stem and progenitor cells (HSPCs) that differentiate into immature myeloid cells (IMCs). According to the model proposed by Gabrilovich et al. (2009), a “two-signal model” explains the molecular mechanisms of MDSC generation and homing in chronic inflammation contexts like cancer [28]. N. Karin further developed this concept, introducing a multistep process including myelopoiesis, mobilization into the bloodstream, homing to the primary tumor site in response to specific cytokines, and hypothetical retention at the tumor site [29]. Two complementary signals allow for the expansion of IMCs and inhibition of their differentiation as well as the activation of immunosuppressive functions.

MDSCs, as their name indicates, exert immunosuppressive activities that severely compromise anti-cancer immunity. MDSC-mediated immunosuppressive mechanisms primarily impair effector antitumor CD4^+^ and CD8^+^ T cells. These suppressive mechanisms include [29,30,31,32]

- the production of reactive oxygen and nitrogen species (ROS and RNS), leading to T-cell apoptosis and proliferation inhibition;
- depletion of essential metabolites for T-cell functions (e.g., arginine and cysteine) leading to their exhaustion and/or apoptosis;
- expression of immune checkpoint ligands, mainly PD-L1 and B7, and apoptosis-inducing ligands such as FasL;
- induction of immunosuppressive cells like regulatory T cells/Tregs and M2 TAMs;
- impaired DC activation (tolerogenic DCs).

Additionally, MDSCs are also endowed with “non-immunological” tumor-promoting properties [33]. They play an essential role in local cancer invasion by modifying the extracellular matrix and fostering migration to adjacent tissues. MDSCs also promote neo-angiogenesis within the primary tumor bed by secreting factors such as MMP9, TGF-β, bFGF, and VEGF, enhancing the permeability of aberrant neo vessels and facilitating tumor cell extravasation, a crucial step in metastatic dissemination [34]. Moreover, MDSCs contribute to pre-metastatic niche formation at distant sites, preparing the “soil” for metastatic tumor cell seeding [35]. Finally, MDSCs are involved in drug resistance in cancer and have been implicated in limiting the effects of chemotherapy and radiotherapy [36].

Recent studies have also shown that MDSCs can promote epithelial-to-mesenchymal transition (EMT) in tumors, thus potentially conferring stem-like properties to cancer cells [37].

This review focuses on research regarding MDSCs in bladder cancer and their potential implications as biomarkers and therapeutic targets.

## 6. Correlation Between MDSCs and BC Stage

The correlation between MDSCs and tumor stage in BC has been investigated by Mu et al. (2019), Yang et al. (2017), and Zhang et al. (2016), revealing significant associations between MDSC accumulation and disease progression [38,39,40].

### 6.1. Correlation in Peripheral Blood

Yang et al. studied the correlation between peripheral blood MDSCs and tumor stage. Their findings indicate that the proportion of *total MDSCs* (*T-MDSCs*) in the peripheral blood of BC patients was markedly higher than that in healthy donors, with a significant elevation in patients at advanced stages T2–T4 compared to those at early stages Ta–T1 (*p* < 0.05). The suppressive capabilities of these *CD11b^+^CD33^+^HLA-DR^−^CD3^−^* cells (ability to suppress T-cell proliferation) was confirmed. Furthermore, Yang et al. observed that the elevated frequency of blood *T-MDSCs* was associated with tumor grade. Zhang et al. corroborated these findings, also reporting a markedly higher proportion of CD33^+^CD11b^+^HLA-DR^−^ and CD33^+^CD11b^−^HLA-DR^−^ *T-MDSCs* in the peripheral blood of BC patients compared to healthy donors [39].

### 6.2. Correlation in Tumor Tissue

In three separate and independent studies, Mu et al., Yang et al., and Zhang et al. evaluated the correlation between tissue MDSCs and tumor stage (Table 2). Mu et al. examined 81 newly diagnosed BC patients (T1 to T4) without neoadjuvant therapy and highlighted the significant role of *M-MDSCs* (CD11b^+^CD45^+^CD33^+^CD14^+^CD15^−^) in the BC immune environment. The authors observed a stage-associated increase (III–IV > I–II). When these *M-MDSCs* were co-cultured with CD8^+^ T cells, a significant inhibition of T-cell proliferation was observed [40]. Similarly, Yang et al. found a substantial increase in *T-MDSC* (*CD11b^+^CD33^+^HLA-DR*^−^*CD3*^−^) accumulation in tumors at stages T2-T4 compared to stages Ta-T1 (*p* < 0.01) [38]. However, it is important to note that the MDSCs characterized by Yang et al. could also represent *PMN-MDSCs* rather than strictly *T-MDSC* due to the markers used (CD11b and CD33 low). Zhang et al. further confirmed a positive correlation between high levels of tumor-infiltrating *T-MDSCs* (CD33^+^CD11b^+^HLA-DR^−^) and advanced disease stage (*p* < 0.05), with the percentage of *T-MDSCs* significantly higher in the tumor tissues compared to the peripheral blood of BC patients [39].

The difficulty in accurately characterizing and identifying MDSC populations underlines the lack of consensus in their phenotypic markers within the scientific community in the phenotypic definition of MDSCs. Nevertheless, these findings suggest a crucial role of MDSCs in BC progression, providing promising avenues for the development of targeted therapeutic strategies.

## 7. Association Between MDSCs and BC Prognosis

The correlation between MDSCs and BC prognosis has been investigated by several researchers, revealing significant associations between MDSC (M-MDSCS, total-MDSCs) accumulation and prognosis at different stages of the disease (Table 3) [38,39,41,42,43,44,45].

### 7.1. Correlation in Peripheral Blood

A study by Fallah et al. involving 109 patients with non-metastatic urothelial carcinoma (UC), regardless of prior treatment, revealed that *UnC-MDSCs* (CD33^+^HLA-DR^−^CD14^−^CD15^−^) were the dominant subtype in whole blood (46%), while *PMN-MDSCs* (CD33^+^HLA-DR^−^CD14^−^CD15^+^) prevailed in peripheral blood mononuclear cells (PBMC) at 41%. Lower levels of *T-* (CD33^+^HLA-DR^−^) and *PMN-MDSCs* were significantly associated with pathological complete response (pCR) following NAC, emphasizing their potential as predictive markers. Conversely, higher levels of *T-, PMN-, M-,* and *UnC-MDSCs* were associated with shorter recurrence-free survival (RFS) and overall survival (OS), as shown by both univariate and multivariate analyses [41].

Ornstein et al. investigated 85 UC bladder patients undergoing cystectomy (regardless of neoadjuvant therapy) and observed a dominance of *UnC-MDSCs* (CD33^+^HLA-DR^−^CD14^−^CD15^−^) in the blood samples. Importantly, lower levels of *T-* (1.8 vs. 3.9%; *p* = 0.006) and *PMN-MDSCs* (0.2 vs. 0.8%; *p* = 0.01) in the blood were associated with a higher likelihood of achieving pCR in multivariate analysis, a well-established positive indicator for improved progression-free survival (PFS) and OS post-cystectomy [42].

In metastatic UC, Sheng et al. reported that *PMN-MDSCs* (CD33^+^HLA-DR^−^CD14^−^CD15^+^) were the leading subtype in both whole blood and PBMC. Their analysis showed that higher levels of *UnC-MDSCs* were significantly associated with a worsening OS (HR 3.78, [1.6–8.8], *p* = 0.002), underscoring their potential as an independent prognostic factor in advanced disease [43].

In a study involving 113 bladder cancer patients, Yang et al. identified *T-MDSCs* as an independent prognostic marker. Their multivariate analysis confirmed that elevated *T-MDSC* levels had a significant impact on OS (HR 3.6, 95% CI [1.5–8.8]; *p* = 0.006) reinforcing the value of *T-MDSCs* as a valuable prognostic tool for assessing BC outcomes [38].

Moreover, Yang et al. showed that in patients with MIBC treated with NAC, higher levels of *PMN-MDSCs* (CD33^+^HLA-DR^−^CD14^−^CD15^+^) were linked to both a significant pathological response (<ypT2) (*p* < 0.001) and better long-term outcomes, such as PFS (HR 4.5, 95% CI [1.9–10.5]; *p* < 0.001) and disease-specific survival (DSS) (HR 6.2, 95% CI [2.7–14.2]; *p* < 0.001), further emphasizing the prognostic relevance of this MDSC subtype [44].

### 7.2. Correlation in Tumor Tissue

Zhang et al. conducted a comprehensive study involving 97 newly diagnosed BC patients who had not received preoperative treatment. By analyzing both fresh and paraffin-embedded tumor tissues, the authors explored the role of tumor-infiltrating MDSCs in BC prognosis. Their findings revealed a significant inverse correlation between the number of tumor-infiltrating *T-MDSCs* and OS (HR 1.9, 95% CI [1.1–3.8]; *p* = 0.04) in a multivariate analysis [39].

### 7.3. Correlation in Urine

Chevalier et al. conducted a phenotypic analysis of immune cells in urine samples from 28 patients with NMIBC undergoing a standard 6-week course of intravesical BCG therapy. Urine samples were collected both before and 4 h following each instillation. As expected, neutrophils were the predominant cell type, but T cells and monocytic cells were also detected. Notably, a significant proportion of the CD14^+^ cells in the urine displayed an *M-MDSC* phenotype (CD11b^+^CD45^+^CD33^+^CD14^+^CD15^−^). Patients with a low T/M-MDSC ratio had a significantly shorter recurrence-free survival (RFS) of 12.7 months compared to 32.7 months in those with a high ratio (*p* < 0.0001). Furthermore, a significant impact on progression-free survival (PFS) was observed (*p* = 0.01). This study suggests a potential link between BCG resistance and type 2 innate lymphoid cells (ILC2), which may enhance the recruitment and immunosuppressive functions of MDSCs via IL-13, highlighting the complex interplay between immune cells and the potential role of MDSCs in BCG therapy resistance [45].

In summary, these studies further emphasize the diverse roles and implications of MDSCs in the different stages and treatments of bladder cancer. Taken together, these data suggest the potential importance of MDSCs as prognostic biomarkers for BC.

## 8. Interaction Between Bladder Cancer Treatments and Myeloid-Derived Suppressor Cells (MDSCs)

### 8.1. BCG

Several studies have demonstrated that BCG can influence MDSC dynamics in bladder cancer, although the effects are context-dependent [46,47].

For instance, Muthuswamy et al. found that BCG treatment increased the expression of chemokines like CXCL8 and CCL22 in BC tissues, which attract suppressive MDSCs and Treg cells, without significantly enhancing CD8^+^ T-cell-attracting chemokines such as CXCL10 [48]. This suggests that BCG may contribute to an environment that supports MDSC accumulation, potentially limiting its therapeutic efficacy. In addition, Takeda et al. found no significant induction of MDSCs in patients undergoing BCG therapy, suggesting that the effect of BCG on MDSC accumulation may vary depending on the specific immune context or treatment conditions [49].

In contrast with these observations, Wang et al. studied the effect of intravesical BCG administration to Sprague-Dawley rats that had developed endogenous orthotopic bladder cancers following their exposure to the carcinogen N-methyl-N-nitrosurea (MNU). They reported that while intravesical BCG treatment alone upregulated PD-L1 expression in bladder cancer cells, potentially reducing its effectiveness, the combination of BCG with systemic anti-PD-L1 antibodies significantly reduced *PMN-MDSC* (CD11b^+^/Gr-1^+^) levels in the tumor microenvironment, thereby enhancing the antitumor immune response [50]. Similarly, Huang et al. also observed a dose-dependent reduction in *PMN-MDSC* (CD11b^+^/Gr-1^+^) levels in C3H mice with orthotopically implanted MBT2 BC treated with high-dose BCG, which led to robust antitumor effects and improved survival [51].

These findings highlight the complex and sometimes contradictory effects of BCG on MDSCs in BC, underscoring the need for further research to determine the real impact of BCG on MDSCs.

### 8.2. Chemotherapy

Chemotherapy has been increasingly recognized for its potential immunomodulatory effects, which extend beyond its traditional role in direct tumor cytotoxicity. According to Emens et al., certain chemotherapeutic agents such as cisplatin, gemcitabine, and paclitaxel can modulate the immune system by enhancing tumor antigen presentation, reducing immunosuppressive cells such as MDSCs and Tregs, as well as promoting the activation and infiltration of effector T cells into the tumor microenvironment [52]. These immunomodulatory properties may synergize with immunotherapies, potentially improving treatment outcomes in cancer patients.

#### 8.2.1. Cisplatin

Cisplatin has been shown to affect MDSCs in bladder cancer, with several studies highlighting its potential to reduce MDSC levels and alter their immunosuppressive capabilities.

Wu et al. studied the effects of in vitro cisplatin administration on PBMC isolated from patients with BC and observed a decrease in *PMN-MDSC* (CD33^+^CD11b^+^CD14^−^CD15^+^) numbers and immunosuppressive function [53]. Indeed, they found that cisplatin-pretreated *PMN-MDSCs* had a diminished ability to suppress CD8^+^ T-cell proliferation.

Similarly, Mu et al. found that intra-arterial infusion chemotherapy (IAIC) using cisplatin in patients with BC reduced *MDSC* levels and enhanced CD8^+^ T-cell activity, thereby decreasing the immunosuppressive environment within the tumor [54].

These findings highlight the consistent immunomodulatory effects of cisplatin on MDSCs in bladder cancer, underscoring the need for further research to fully understand its impact on the tumor microenvironment.

#### 8.2.2. Gemcitabine

Current evidence regarding the impact of gemcitabine on MDSCs in BC derives exclusively from mouse models, with two studies showing contradictory results regarding the effects of this chemotherapy on MDSC recruitment and function within the tumor microenvironment.

Mu et al. found that gemcitabine not only inhibited the proliferation of bladder cancer cells but also increased the recruitment of *M-MDSCs* [40]. This was linked to elevated levels of CCL2, both at the transcriptional and protein levels, in bladder cancer cell lines treated with gemcitabine. The use of RS 504393, a selective CCL2 receptor antagonist, in these models significantly reduced M-MDSC recruitment and improved survival by blocking their chemotaxis.

In contrast, Bazargan et al. investigated the use of gemcitabine in combination with adoptive cell therapy (ACT) in mice with bladder cancer [55] and showed that gemcitabine could deplete MDSCs within the tumor microenvironment, thereby enhancing the efficacy of ACT. Their murine model revealed that MDSCs were the predominant immune cells within the tumor microenvironment, with PMN-MDSCs being particularly prevalent. Intravesical gemcitabine treatment led to significant depletion of immune cells, including MDSCs, which correlated with reduced tumor growth when combined with ACT.

### 8.3. Systemic Immunotherapy: Immune Checkpoint Inhibitors (Anti-PD-1/PD-L1)

Takeyama et al. explored the role of MDSCs and their interaction with (ICI) in a cisplatin-resistant bladder cancer model using the MB49 xenograft model in mice [56]. They found that *M-MDSCs* were more prevalent in tumors and were more strongly associated with tumor progression compared to *PMN-MDSCs*. In cisplatin-resistant tumors, *M-MDSCs* were found to infiltrate more frequently, contributing to the observed resistance. Importantly, the study showed that blocking MDSCs using anti-Gr1 and anti-Ly6C antibodies, in combination with anti-PD-L1 antibodies, significantly enhanced antitumor effects, as evidenced by reduced MDSC levels and increased CD8^+^ T-cell infiltration without adverse immune events. These findings suggest that co-targeting MDSCs and PD-L1 could improve the efficacy of immunotherapy in cisplatin-resistant bladder cancer. However, it is important to note that Gr1 and Ly6C are mouse-specific markers of MDSCs, while identifying equivalent markers in humans is more challenging due to their higher number and lower specificity. Furthermore, the combination of such therapies is known to increase the risk of toxicity, thus posing a greater concern regarding their application in clinical settings.

In contrast, clinical data from Tzeng et al. evaluated the modification of MDSCs and CD8^+^ T cells in response to ICI in metastatic UC patients [57]. They found that treatment with anti-PD-L1 agents (avelumab or atezolizumab) correlated with a decrease in PD-L1^+^ *M-MDSCs* (CD33^+^ CD11b^+^ HLA-DR^−^ CD14^+^ CD15^−^), while anti-PD-1 treatment (pembrolizumab) was associated with a decrease in PD-1^+^ *M-MDSCs*. However, these changes in PD-L1/PD-1 expression by MDSC subsets did not predict treatment response in univariate analysis, and no correlation was found between pre- and post-ICI expression of PD-1/PD-L1 by MDSCs and clinical outcomes. Finally, Wang et al. investigated the effects of BCG treatment on PD-L1 expression in BC cells and also evaluated the efficacy of BCG and anti-PD-L1 combination therapy in immunocompetent orthotopic rat BCa models [50].

They found that PD-L1 expression was obviously upregulated in BCa cells in response to BCG treatment both in vitro and in vivo. Moreover, BCG and anti-PD-L1 combination treatment activated a potent antitumor immune response with the increase in the number and activity of tumor-infiltrating CD8^+^ T cells, as well as the reduction in M-MDSCs.

### 8.4. Radiation Therapy

Yamamoto et al. explored the mechanism of overcoming treatment resistance in immunologically cold tumors by combining radiation therapy (RT) with MDSC-targeting therapy [58]. Their study, using MB49 and cisplatin-resistant MB49R mouse models, revealed significant differences in the tumor microenvironment between the two, with higher levels of CD8^+^ T cells and *PMN-MDSCs* (CD11b^+^Ly6C^low^Ly6G^+^) in MB49 tumors compared to MB49R tumors. Additionally, PD-L1 expression was notably higher in MB49 tumors, making MB49R tumors appear immunologically colder. Flow cytometry analysis showed that *PMN-MDSCs* increased rapidly after both conventional and hypo-fractionated RT, while CD8^+^ T cells increased only in MB49 tumors. Combining RT with MDSC-targeting therapy (anti-Ly6C and anti-Gr1 antibody) significantly reduced tumor growth in both MB49 and MB49R models, and the combination was more effective than either treatment alone. Specifically, the reduction in tumor size was more pronounced when RT was combined with MDSC blockade compared to monotherapies, particularly in the cisplatin-resistant MB49R model. This study suggests that PMN-MDSCs are an ideal target when combined with RT, offering a promising strategy to overcome resistance in immunologically cold tumors.

## 9. Specific Targeting of MDSCs in Bladder Cancer and Perspectives

MDSCs play a pivotal role in dampening the immune response against tumors, thus promoting cancer progression. Numerous preclinical studies have tested strategies for targeting MDSCs across various cancers, employing approaches such as depleting MDSCs, blocking their recruitment, inhibiting their suppressive activity, and inducing their differentiation into non-immunosuppressive myeloid cells [59,60,61,62].

### 9.1. Depleting

Chemotherapies, such as gemcitabine and cisplatin, have been shown to selectively deplete MDSC populations in BC [50,53], while tyrosine kinase inhibitors, such as sunitinib, have demonstrated efficacy in depleting MDSCs in renal cancer [63].

### 9.2. Blocking Recruitment of MDSCs

Blocking the recruitment of MDSCs to the tumor microenvironment has been attempted using chemokine inhibitors that target pathways such as CCR5 (Maraviroc) and CXCR2 (Reparixin) in phase 1/2 clinical trials for metastatic colorectal cancer (NCT 01736813) and breast cancer (NCT 02370238). Preliminary data from Wu et al. of RS 504393 (selective CCL2 receptor antagonist) showed reduced M-MDSC recruitment and improved survival of BC patients [40]. Other preliminary results from Zhang et al. found that the inhibition of CXCR2 is crucial for MDSC recruitment in BC, and its inhibition could improve patient prognosis [39].

### 9.3. Inhibit MDSC Activity

Additionally, inhibitors of STAT3, COX-2, and PDE5 have been tested to reduce MDSC immunosuppressive activity, showing potential in cancers, such as melanoma and colorectal cancer. In BC, Prima et al. highlighted that the COX-2/mPGES1/PGE2 pathway increases PD-L1 expression in MDSCs, promoting immune suppression. Inhibiting this pathway with COX-2 inhibitors such as celecoxib significantly reduces immune suppression in the tumor microenvironment [64]. Furthermore, Zheng et al. suggested that in patients with MIBC, IL-6 promotes the immune suppression ability of MDSCs by activating the MAPK signaling pathway [65]. Inhibition of this pathway using TOCILIZUMAB (an IL-6 inhibitor) could be an interesting perspective.

### 9.4. Differentiating MDSCs

Furthermore, differentiation strategies using compounds, such as all-trans retinoic acid (ATRA) or taxane-based chemotherapy (docetaxel), have been used to convert MDSCs into mature myeloid cells that no longer suppress the immune system. However, none of these therapies have been tested for BC.

Despite these advances, it is important to note that there are currently no clinical trials that specifically target MDSCs in BC. Most BC studies have focused on immune checkpoint inhibitors or conventional chemotherapies, leaving a gap in understanding how MDSC-targeted therapies might benefit this specific cancer type. Further research is needed to explore how these therapies, effective in other cancers, could be applied to improve outcomes in bladder cancer.

## 10. Conclusions

The role of MDSCs in bladder cancer has emerged as a potential factor influencing disease progression and therapeutic resistance. Studies have consistently demonstrated that MDSCs, through their immunosuppressive functions, contribute to the ability of tumors to evade immune detection and hinder the efficacy of treatments. While targeting MDSCs in preclinical models has shown promising results, translating these findings into effective clinical therapies remains a challenge due to the difficulty in identifying equivalent markers in humans and the increased risk of toxicity when combining multiple therapies. Future research should focus on refining these therapeutic approaches, developing better biomarkers to identify MDSC subsets, and exploring combination strategies that target both intrinsic and microenvironmental factors to improve patient outcomes in bladder cancer.

## Figures and Tables

**Table 2:** Summary of studies investigating the association between MDSC subsets and bladder cancer stage.

Study	*n*	BC Stage	Localization	MDSC Subset	Association
Mu et al. (2019) [40]	81	T1-T4	Tissue	M-MDSCs CD11b^+^CD45^+^CD33^+^CD14^+^CD15^−^	Stage
Yang et al. (2017) [38]	113	Ta-T4	Blood and Tissue	T-MDSCs CD11b^low^CD33^low^HLA-DR^−^CD3^−^	Stage and tumor grade
Zhang et al. (2017) [39]	108	Ta-T4	Blood and Tissue	T-MDSCs CD33^+^CD11b^+^HLA-DR^−^	Stage

**Table 3:** Summary of studies investigating the association between MDSC subsets and bladder cancer prognosis.

Study	*n*	MIBC/NMIBC	Localization	MDSC Subset	Association
Fallah et al. (2020) [41]	109	MIBC	Blood	Low T-MDSCs PMN-MDSCs	pCR (ypT0)
				High T-MDSCs PMN-MDSCs M-MDSCs UnC-MDSCs	RFS and OS
Ornstein et al. (2018) [42]	85	MIBC	Blood	Low T-MDSCs PMN-MDSCs	pCR, PFS, and OS
Sheng et al. (2020) [43]	76	MIBC	Blood	High UnC-MDSCs	OS
Yang et al. (2017) [38]	113	MIBC	Blood	High T-MDSC	OS
Yang et al. (2020) [44]	91	MIBC	Blood	Low PMN-MDSCs	PR (<yPT2)
					PFS and DSS
Zhang et al. (2017) [39]	97	NMIBC & MIBC	Tumor tissue	High T-MDSC	OS
Chevalier et al. (2017) [45]	28	NMIBC	Urine	low T/M-MDSC	RFS and PFS

MIBC = muscle-invasive bladder cancer; NMIBC = non-muscle-invasive bladder cancer; pCR = pathological complete response; RFS = recurrence-free survival; OS = overall survival; PR = pathological response; DSS = disease-specific survival; PFS = progression-free survival.

## Data Availability

Not applicable.
